# The Use of Hydrogel-Based Materials for Radioprotection

**DOI:** 10.3390/gels9040301

**Published:** 2023-04-03

**Authors:** Yang Li, Han Liu, Yaqun Ding, Wanyu Li, Yuansong Zhang, Shenglin Luo, Qiang Xiang

**Affiliations:** 1 Center of emergency, First Affiliated Hospital, Third Military Medical University (Army Medical University), Chongqing 400038, China; 2 Institute of Combined Injury, State Key Laboratory of Trauma, Burns and Combined Injury, Chongqing Engineering Research Center for Nanomedicine, College of Preventive Medicine, Third Military Medical University (Army Medical University), Chongqing 400038, China

**Keywords:** hydrogels, radiation injury, radioprotection

## Abstract

Major causes of the radiation-induced disease include nuclear accidents, war-related nuclear explosions, and clinical radiotherapy. While certain radioprotective drug or bioactive compounds have been utilized to protect against radiation-induced damage in preclinical and clinical settings, these strategies are hampered by poor efficacy and limited utilization. Hydrogel-based materials are effective carriers capable of enhancing the bioavailability of compounds loaded therein. As they exhibit tunable performance and excellent biocompatibility, hydrogels represent promising tools for the design of novel radioprotective therapeutic strategies. This review provides an overview of common approaches to radioprotective hydrogel preparation, followed by a discussion of the pathogenesis of radiation-induced disease and the current states of research focused on using hydrogels to protect against these diseases. These findings ultimately provide a foundation for discussions of the challenges and future prospects associated with the use of radioprotective hydrogels.

## 1. Introduction

Steady advances in nuclear technologies have led to the increasing modernization of the nuclear arsenals of many nations, driving an intensifying nuclear arms race and contributing to an increasingly tense global security situation [1]. Rates of nuclear accidents are also rising, leading to serious economic losses, casualties, and the pollution of local environments [2]. Radiation is additionally deployed as an antineoplastic treatment in many cancers in combination with chemotherapy and surgery, with over half of all cancer patients ultimately requiring some degree of radiotherapy. While the high-energy ionizing radiation employed when administering radiotherapy can effectively kill tumor cells, it can also cause serious damage to surrounding healthy tissue, thereby causing potentially debilitating adverse effects [3,4].

Ionizing radiation can cause both early adverse events and late chronic effects in exposed healthy tissues. Early radiation-induced reactions include mucositis, dermatitis, reductions in blood cell counts, and pneumonia. At later time points, adverse outcomes can include bone tissue necrosis, vascular damage, tissue fibrosis, nerve injury, and the radiation-induced development of secondary malignancies. The generally irreversible nature of these delayed complications can pose a particularly serious long-term risk to patients [3,4].

Advances in the design of radioprotective agents are critical to improving radiotherapy outcomes by minimizing harm to normal tissues. While several candidate radioprotectors have been synthesized to date, the majority are organic compounds limited by poor solubility in water, rapid clearance from systemic circulation, and rapid metabolic processing. As such, their pharmacological efficiency is very poor [5,6]. In addition, biological active ingredients such as mesenchymal stem cells (MSCs) and cytokines that can hasten the repair of radiation damage are also tried [7,8]. However, in most clinical studies to date, MSCs have been delivered in liquid suspensions that contribute to suboptimal cell retention and underwhelming therapeutic efficacy [9]. It is thus critical that new approaches to enhancing the bioavailability of these agents or replacing them with more effective compounds are established.

Tissue engineering strategies rely on the utilization of biomedical engineering and biological methods to restore the morphology and function of injured organs or tissues [10]. These strategies can be adapted to treat radiation-induced tissue damage. Hydrogels, serving as a 3D polymer network rich in water, can provide a humid environment [11], which is conducive to the wound recovery and regeneration of damaged tissues caused by radiation damage. The structure of hydrogel is usually similar to that of extracellular matrix, showing good histocompatibility [12]. Taking advantage of this feature, it can serve as a carrier for radioprotective drug such ROS scavenger [13]. These hydrogels can persist on tissue surfaces for extended periods of time, thereby stably releasing their cargos and enhancing their bioavailability [14]. Growth factors, stem cells, and other substances of interest can also be loaded into hydrogels [15,16,17]. The structurally similar to human soft tissues and tunable physiochemical properties also make them promising tools for the radionuclide decontamination of the skin and the isolation of radiotherapy-target tissues [18,19]. In addition, the stimulus response hydrogel, whose volume or structure is changed according to the change of external conditions (temperature, pH or light, etc.), attracts more attention in different areas [20,21]. According to the characteristics of ROS excess production in radiation injury [22], ROS-responsive hydrogel also provides a new idea for radiation damage treatment. Accordingly, in this article, we summarize common approaches to develop hydrogels for preventing or treating damage caused by ionizing radiation. We then focus on the radioprotective effects of these hydrogels for a range of tissues and organs while also discussing the pathogenesis of radiation-induced disease. Challenges and future opportunities for radioprotective hydrogel design are also discussed. The overall goal of this review is to provide a clearer understanding of the current status of research focused on hydrogel-mediated radioprotection in an effort to spur further advances in this research field.

## 2. Approaches to the Design of Radioprotective Hydrogels

The choice of materials largely determines the performance of hydrogels [23]. Compared with synthetic ingredients, natural ingredients, which come from biology, have good biocompatibility and are easy to be degraded by enzymes [24]. They are usually well integrated into biological tissues, and thus are first considered as materials for radiation protection hydrogels [25]. Among them, polysaccharides such as alginate, chitosan, and hyaluronic acid (HA) are particularly popular. Polysaccharides have some excellent properties, such as non-toxicity, biocompatible, biodegradable, bio-stable, abundant, and susceptible of enzymatic digestion in the human body [25]. In addition to polysaccharides, gelatin obtained by collagen hydrolysis and ECM prepared by acellular allogeneic tissues/organs have also been selected by designers as materials for radiation protection hydrogels due to their excellent cell adhesion and biocompatibility [26,27].

**Figure 1:**
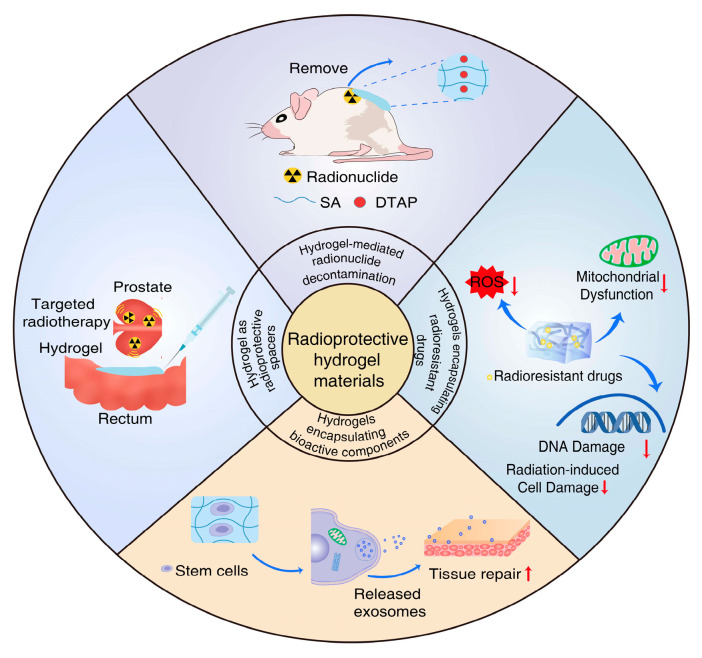
Scheme of the general hydrogel materials for radiation protection.

Various types of hydrogels have been assessed for radioprotective efficacy in basic experiments or clinical settings. Four major approaches to the design of radioprotective hydrogels include hydrogels used for radionuclide decontamination, hydrogels encapsulating radioresistant drug, hydrogels encapsulating bioactive components, and the use of hydrogels as radioprotective spacers (Figure 1).

### 2.1. Hydrogel-Mediated Radionuclide Decontamination

When attacks or accidents expose individuals to radionuclides, affected individuals may experience injury and contamination with radionuclides capable of entering systemic circulation and specific tissues through wounds. This, in turn, can result in prolonged internal organ exposure to irradiation, posing a major hazard to human health. Prolonged high-dose radionuclide exposure can also have adverse outcomes including cancer [28]. Rapid decontamination of skin wounds following radionuclide exposure is thus essential. The traditional decontamination strategy entails the use of saline to wash contaminated sites in vitro, but this can cause the contaminants to diffuse into surrounding sites owing to the fluid nature of the utilized saline [29]. To overcome these issues, several research teams have developed hydrogels designed to facilitate radionuclide decontamination, thereby protecting against radiation-induced damage. For example, Cui et al. [30] utilized the physical cross-linking of polyacrylamide, sodium alginate (SA), and diethylene triamine pentaacetic acid (DTPA) through hydrogen bonding to generate a poly(AAm-SA-DTPA) (PASD) hydrogel. This PASD hydrogel material exhibited promising adhesive and mechanical properties without causing cytotoxicity. Owing to the ability of DTPA and SA to absorb radionuclides, it was also able to effectively decontaminate skin wounds and to facilitate improved wound healing. This hydrogel is thus a viable candidate for use when seeking to decontaminate radionuclide-exposed skin wounds. Xu et al. [31] similarly designed bisphosphonate-containing supramolecular hydrogels through the incorporation of a uranium chelating agent. Experimental results in mice showed that the hydrogel could remove uranium ions from contaminated wounds effectively.

### 2.2. Hydrogels Encapsulating Radioresistant Drug

**Figure 2:**
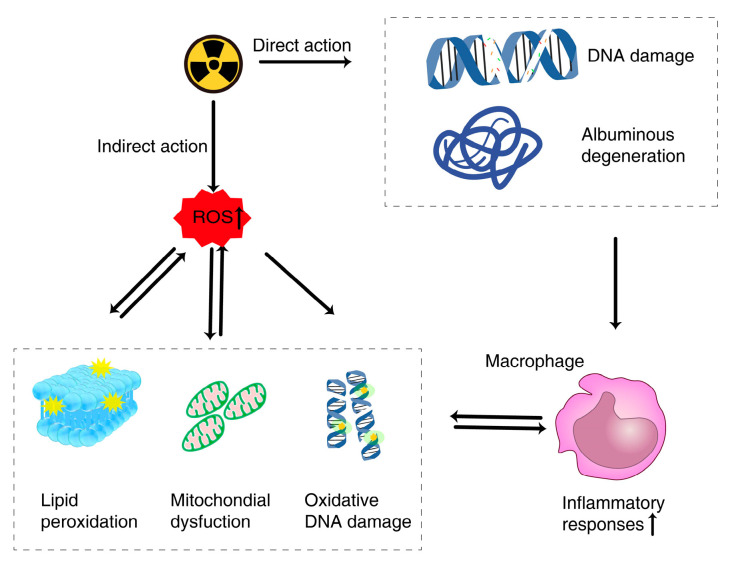
Mechanisms of radiation-induced injury.

Radioresistant drug can prevent or mitigate radiation-induced damage when administered either prior to or following radiation exposure. Mechanistically, ionizing radiation can cause damage through the infliction of direct damage to DNA and proteins. In addition, it can indirectly damage a range of biomolecules by producing an overabundance of reactive oxygen species (ROS) including superoxide anion (O2^•−^), hydrogen peroxide (H_2_O_2_), singlet oxygen (^1^O_2_), and hydroxyl radical (^•^OH) when interacting with water molecules. These excessively high ROS levels can promote oxidative stress, resulting in the mitochondrial dysfunction, lipid peroxidation, oxidative DNA damage, and ultimately cause cell death [32,33]. Furthermore, inflammation is also confirmed to be involved in the development of radiation-induced injury [34] (Figure 2). Protecting against radiation-induced damage thus centers around repairing the DNA, scavenging free radicals and anti-inflammatory [35]. Many radioresistant classes of radioprotective drugs have been developed to date including chemical drug (aminothiol, indomethacin, fullerenol, etc.), biologics (superoxide dismutase [SOD], SOD mimetics, etc.), and herbal compounds (curcumin [Cur], cordycepin, etc.) [5,6,35]. Of these, fullerenol has been leveraged as a free radical sponge capable of protecting the skin from radiation-induced damage, exhibiting excellent biosafety and chemical stability in addition to its robust ROS scavenging ability [36]. Zhao et al. [37] designed a fullerenol-enriched medical sodium hyaluronate hydrogel, and found that it could effectively protect epidermal stem cells, thereby alleviating radiation-induced dermatitis when applied to the skin. Zhang et al. [38] also generated a Cur- and epigallocatechin gallate (EGCG)-encapsulating hydrogel that protected against radiation-induced skin lesion formation through ROS scavenging, the induction of angiogenesis, and anti-inflammatory activity. 

### 2.3. Hydrogels Encapsulating Bioactive Components

A variety of bioactive compounds and cells including stem cells and growth factors can be readily loaded into hydrogels owing to the fact that they are prepared under mild conditions [39,40]. Such hydrogels have successfully been leveraged for the treatment of radiation-induced damage. Nie et al. [41], for example, were able to encapsulate MSCs derived from gingival tissue in self-assembling peptide hydrogels. When they deployed these hydrogels in vivo, they effectively mediated the enhanced healing of skin tissue following irradiation. The extracellular matrix (ECM) is composed of a variety of proteins that provide a physical matrix with which cells can interact, in addition to functioning as bioactive ligands. The ECM contains a range of proteoglycans, fibrous structural proteins, and growth factors that can support the repair of injured tissues. Zhang et al. [42] generated a lung ECM-derived hydrogel that was capable of alleviating pulmonary damage and edema following radiation exposure, while Dong et al. prepared an esophagus-derived ECM hydrogel that reduced the severity of esophageal inflammation upon radiation exposure. Platelet-rich hydrogels can also reportedly protect against radiation-induced bone injury and dermatitis, leading to growing interest in their clinical application [43,44].

### 2.4. Hydrogels as Radioprotective Spacers

The accuracy of clinical radiotherapy has been augmented by the development of imaging-guided dosing strategies. Even so, achieving sufficient space between the target tumor and healthy tissue to ensure that no unintended damage occurs is generally impossible owing to the inevitable proximity between the two [3]. In an effort to maximize the radiotherapy dose delivered to tumors while preserving adjacent tissue integrity, clinicians use spacers to adjust the relative positions of tumors and healthy organs [45]. In this context, hydrogel shims offer advantages including excellent histocompatibility and absorbability. The radiotherapeutic treatment of prostate cancer patients, for example, is complicated by dose limitations associated with the proximity between the prostate and the rectum. A hydrogel can be implanted prior to treatment between the rectum and the prostate, thus increasing the radiation dose delivered to the prostate while minimizing rectal injury [46,47]. Similar hydrogel-based spacer strategies have also been employed for the radiotherapeutic treatment of head and neck cancer and cervical cancer patients [48,49].

## 3. The use of Hydrogels for the Treatment of Radiation-Related Disease

Researchers have successfully leveraged a range of hydrogel-based radioprotective materials using the strategies detailed above in recent years, highlighting the clinical promise associated with their application. In this section, we explore the use of hydrogels to date in an effort to treat or prevent various radiation-related diseases affecting particular organs and tissues.

### 3.1. Radiation-Induced Skin Injury

External beam radiotherapy strategies inevitably expose the skin to high doses of ionizing radiation which passes through this tissue to reach the target tumor site. As such, radiation-induced skin injury (RISI) is the most prevalent form of radiation-related disease, with all radiotherapy patients being at risk for this condition. RISI rates are particularly high for patients with tumors in the breast, chest wall, skin fold, and head and neck regions. RISI only manifests multiple days after treatment initiation, causing both acute and chronic symptoms. Early acute symptoms can include dry desquamation and generalized erythema, skin folds, wet desquamation, and potentially severe ulceration, hemorrhage, and skin necrosis. In contrast, chronic reactions such as radiation-induced keratosis, ulceration, cutaneous fibrosis, telangiectasia, and secondary skin cancer may only manifest months or years following treatment [50,51]. Oxidative stress and inflammation are the main factors that drive skin injury in this context, providing target pathways for the design of viable therapeutic interventions [34].

Pluripotent MSCs are present within many tissue compartments including the spleen, brain, dermal tissue, adipose tissue, and bone marrow. As they are capable of secreting high levels of cytokines, regulating immune function, and differentiating into a range of cell types, MSCs are regarded as important targets in regenerative medicine [52,53]. These MSCs exhibit robust regenerative capabilities in models of tissue injury including diabetes- and burn-related wound model systems, making them promising tools that can be leveraged to treat radiation-related skin damage [54,55,56]. Hydrogels represent an ideal tool for MSC delivery owing to their excellent biocompatibility, biodegradability, and mechanical properties [57]. Lee et al. [58], for example, utilized the ECM-based pig small intestinal submucosa (SIS) for hydrogel preparation, incorporating MSCs derived from human umbilical cord blood (hUCB) into this scaffold. This resulted in enhanced MSCs proliferation and superior exocrine functionality, improving wound healing in mice following hUCB-MSCs/SIS hydrogel composite application. Mechanistically, this combination of MSCs and hydrogel materials was found to secrete hepatocyte growth factor and other pro-angiogenic cytokines, while also recruiting endothelial cells to the regenerating wound site, ultimately contributing to better radiation-related wound healing [59,60]. In another study [41], the Nap-GDFDFDY (Y-Gel) molecular hydrogel, which consists of co-assembled peptide gel and protein, exhibits excellent biocompatibility with murine immune cells. Using ^37^Cs to induce a model of radiation-induced skin injury, researchers subcutaneously injected Gingiva-derived MSCs (GMSCs)-loaded Y-Gel into mice, revealing a significant reduction in the severity of skin wounding as compared to control irradiated mice owing to the ability of this hydrogel and the incorporated GMSCs to promote the repair of damaged cutaneous tissue [41]. This hydrogel can thus allow GMSCs to exert their biological effects, thereby facilitating wound healing. This pY-Gel self-assembling peptide hydrogel or similar GMSC-loaded platforms may hold promise as tools to treat radiation-induced skin injury.

**Figure 3:**
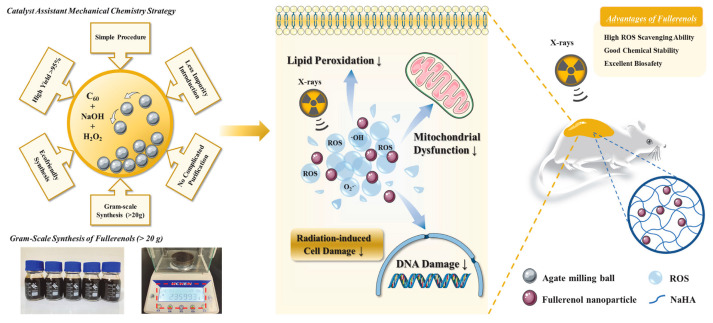
Gram-scale synthesis and protective mechanism of fullerol for skin radiation protection. Reprinted with permission from [37].

Radioprotective hydrogels have also been prepared by incorporating radioresistant chemical compounds. For example, the carbon-based fullerene-derived nanomaterial fullerenol, which is prepared via hydroxylation of the fullerene carbon cage, can readily remove ROS. Zhao et al. [37] generated fullerenol sodium hyaluronate (F-NaHA) hydrogels and applied them for cutaneous radioprotection. The resultant hydrogel exhibited excellent moisture retention, viscoelasticity, and was highly tissue-compatible. When this F-NaHA hydrogel was applied to the skin, the fullerenol therein was able to effectively serve as a broad-spectrum scavenger for free radicals, protecting against ROS-induced tissue damage in mice to preserve the integrity of the epidermal basal layer and the viability of epidermal stem cells, thus alleviating radiation dermatitis and emphasizing the potential protective value of this strategy (Figure 3).

Having all these studies in mind, we summarize the representative hydrogel materials reported on animal models of RISI in Table 1.

There have been reported clinical trials in which hydrogels have been employed to treat RISI patients. In total, 9 studies were identified in which hydrogels were used to treat radiation dermatitis patients, including 7 randomized controlled studies (RCT) and 2 single arm studies (shown in Table 2). Of these, a boron-based hydrogel exhibited the greatest promise, with Aysan et al. [64] having used this gel to treat 47 patients with breast cancer, resulting in reduced RISI as determined with the Radiation Therapy Oncology Group scoring system. Mechanistically, the authors speculated that this protective activity may have been mediated by the thermal degradation, pro-regenerative, and antioxidant effects of boron. Another randomized trial [65] enrolling 257 patients also demonstrated the ability of boron-based hydrogels to prevent most forms of radiation dermatitis in females undergoing radiotherapy to treat breast cancer, alleviating conditions including dermatitis, erythema, dry desquamation, and moist desquamation. Other studies have demonstrated the preventative or therapeutic effects of HA gels, film-forming silica gel, and etc. in the context of radiation dermatitis, although these reports only included a limited number of cases. Hydrogels exhibit clear advantages when treating RISI owing to their ability to adapt to the surface of the skin, maintaining local moisture while cooling the area to improve patient comfort [66]. Despite these advantages, sufficient clinical evidence demonstrating the superiority of boron-based or other hydrogels when managing RISI is lacking at present. As such, further large-scale trials will be vital to confirm the effects of different hydrogel materials and to explore a wide range of hydrogel types incorporating bioactive compounds or other cargos in an effort to improve the clinical management of radiation dermatitis.

### 3.2. Osteoradionecrosis

Radiation can have severe effects both on local bone tissue within the target treatment field as well as a systemic adverse impact, causing complications such as osteopenia, fracture, impaired bone growth, and avascular necrosis. Osteoradionecrosis (ORN) is among the most prevalent forms of severe radiotherapy-induced complications, with jaw osteonecrosis and avascular necrosis of the femoral head being particularly common in patients with head and neck tumors and pelvic tumors, respectively [74]. ORN incidence rates are estimated to range from 3.5 to 4.74%, resulting in a range of symptoms [75]. In the oral and maxillofacial regions, the main symptoms include exposed necrotic bone with wound dehiscence or fistulization, with pathological fractures in severe cases [76]. The precise pathogenic basis for ORN remains to be fully clarified. While one theory posits that radiotherapy causes a range of cellular and extracellular events that result in hypoxia, tissue hypovascularity, and hypocellularity [77]. In contrast, Delanian suggested that ORN is primarily driven through a radiation-induced fibroatrophic model in which radiotherapy results in dysregulated fibroblast activation [78]. Given these uncertainties regarding the pathogenesis of ORN, the most effective means of treating this condition remain uncertain. Conservative treatment strategies include antibiotics, resection, ultrasonic therapy, and hyperbaric oxygen therapy. Surgery is necessary in severe cases, including segmental mandible resection with defect reconstruction as appropriate. Free flap reconstruction approaches can reduce operative durations but can also cause donor site injuries. Even when appropriate postoperative care is implemented, recurrent ORN can often occur [79,80]. A range of treatments for ORN have been proposed including antioxidants, growth factors, and MSCs, with hydrogels holding great promise as a platform for the delivery of these factors to sites affected by ORN [81,82,83].

**Figure 4:**
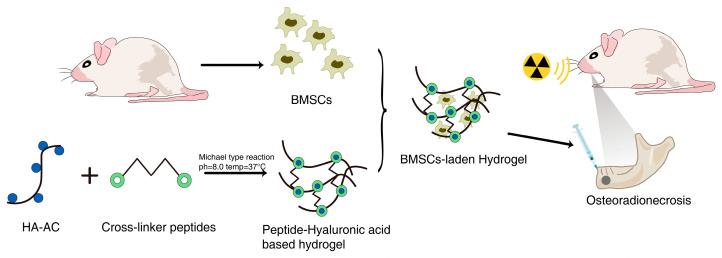
Schematic illustration of synthesis and application of BMSCs-laden hyaluronic acid-based hydrogel.

Studies have employed hydrogels loaded with bone marrow MSCs (BMMSCs), which can differentiate into neurons, osteoblasts, chondrocytes, and adipocytes in an effort to treat ORN. For example, Jin et al. [84] prepared hyaluronic acid hydrogels incorporating rat BMMSCs along with bone morphogenetic protein-2 (BMP-2), which can readily promote the regeneration of bone tissue. This treatment led to significant increases in bone mass (BV) and bone mineral density (BMD) in a mouse model of radiation-induced bone defects. The prepared matrix metalloproteinase (MMP)-sensitive acrylate HA hydrogel was able to simulate the plastic properties of cell-derived MMPs on a natural ECM, improving rates of tissue remodeling and enhancing bone regeneration by delivering BMMSCs and BMP-2 to target sites (Figure 4). Matrigels loaded with tonsil-derived mesenchymal stem cells (TMSCs) were also able to protect against ORN, improving BMD significantly in treated mice at 4 weeks post-modelling relative to control mice. These TMSC-loaded matrigels may thus be able to effectively support ORN bone regeneration, particularly if administered immediately following dentoalveolar trauma or surgery [85].

Several groups have sought to design growth factor-loaded hydrogels. Protein members of the transforming growth factor (TGF)-β superfamily serve as essential mediators that can induce bone growth, maintain bone integrity, and promote bone repair. TGF-β can bind to latency-associated peptides and latent TGF-β binding protein, enabling the storage of this growth factor within the ECM for subsequent use upon bone injury. This latent TGF-β can undergo activation in response to disulfide bond cleavage. Accordingly, the in vivo delivery of active TGF-β necessitates its incorporation into a carrier capable of shielding it from undergoing degradation or interacting with inhibitor molecules in the ECM. Ehrhart et al. [86] explored the preparation of a TGF-β-loaded gelatin gel, which they found was capable of effectively promoting radioactive bone injury repair in rabbits. The prepared bioresorbable gelatin hydrogel exhibited a favorable isoelectric point amenable to TGF-β absorption while forming a barrier that prevented it from interacting with the ECM. Importantly, this barrier layer was susceptible to biochemical manipulation, facilitating the release of appropriate TGF-β concentrations over time, demonstrating the promise of this cytokine carrier platform. Table 3 lists the main data on animal models of ORN treated using hydrogel materials.

Despite extensive preclinical work, only two case reports have described the clinical use of hydrogels to treat ORN [44,87]. In both of these studies, platelet-rich hydrogel was employed as a tool for promoting mandibular necrosis regeneration and associated defects in cancer patients undergoing radiotherapeutic treatment. Over the course of the follow-up, both patients experienced the complete recovery of alveolar bone mass consistent with having been clinically cured. This hydrogel-based approach employed the use of autologous platelet-rich plasma (PRP) that was activated by thrombin and calcium exposure, causing it to form a gel with adhesive and plastic properties that can be effectively employed as a local treatment for damaged tissues. The resultant PRP-based gel can be cut in a range of shapes, making it highly effective for clinical use. PRP-based gel can promote accelerated tissue repair and regeneration mediated by the high levels of growth factors present within platelet alpha granules. While these early results are promising, further randomized controlled trials enrolling larger numbers of patients will be vital to definitively establish the clinical value of platelet-rich hydrogels as treatments for ORN.

### 3.3. Radiation-Induced Damage to Adjacent Healthy Tissues

Prior to undergoing radiotherapy, clinicians can place hydrogel spacers in between the target organ and surrounding healthy tissues, thereby providing patients with clinical benefits [45]. These hydrogel spacers are primarily applied in prostate cancer patients, but they have also been deployed in cervical, breast, and other cancers when radiotherapy is being performed [88,89,90]. A range of injectable hydrogels have been designed using polyethene glycol (PEG), HA, and collagen. In this section, we discuss the relative advantages of these different materials with respect to their characteristics, efficacy, and tolerability when applied as hydrogel spacers.

#### 3.3.1. PEG Hydrogels

The synthetic polymer PEG is widely used when preparing controlled-release systems that seek to take advantage of its hydrophilic, biodegradable, and biocompatible properties [91]. PEG hydrogels are also the most frequently used radioprotective spacers in prostate cancer. Absorbable PEG hydrogels exhibit satisfactory water solubility and histocompatibility and can be injected in a liquid form into a target organ wherein they can remain stable for roughly 3 months, after which they undergo gradual hydrolysis and renal excretion. This absorption process is generally complete within a 12-month period [92,93]. Pinkawa et al. [94] utilized PEG hydrogels in several phase II studies of prostate cancer patients undergoing radiotherapy, enabling the assessment of the spatial and dosing effects of these hydrogels and their ability to shield rectal tissue from high levels of radiation. These hydrogels remained stable during radiotherapeutic treatment, but were completely absorbed after 12 months such that they were undetectable via MRI. When evaluating the 5-year quality of life outcomes of patients injected with these hydrogel spacers, significantly better long-term outcomes including lower rectal toxicity and better intestinal quality of life were evident in patients that received these spacers. While they do entail additional medical costs, the routine use of hydrogel spacers may ultimately lower healthcare costs by mitigating the severity and reducing the incidence of proctitis in individuals undergoing radiotherapy [95].

#### 3.3.2. HA Hydrogels

HA is a natural non-sulfurized glycosaminoglycan that serves as a major ECM component and is ubiquitously present throughout vertebrates. HA exhibits low immunogenicity and excellent biocompatibility [96]. Leveraging these properties, Wilder et al. [97] reported an injectable HA hydrogel for use as a radioprotective spacer in prostate cancer patients undergoing radiotherapy. In addition, the efficacy of HA applications has been confirmed in the context of the radiotherapeutic treatment of both gynecological and mediastinal tumors, although these HA hydrogels exhibit suboptimal stability when exposed to high doses of radiation owing to the tendency of the viscosity and elastic modulus of the gel to decrease in response to irradiation [90,98]. HA is a ligand for the cell surface proteins ICAM-1 (intercellular adhesion molecule-1) and CD44, and signaling through these proteins can induce migratory, proliferative, and invasive activity in cells. This may provide advantages to exposed tumor cells, underscoring the importance of injecting HA-based materials only into the surrounding space without penetrating the tumor compartment [98]. This drawback has the potential to restrict the application of HA-based spacers in the context of radiotherapeutic intervention.

#### 3.3.3. Collagen Hydrogels

Collagen is a natural polymer and a primary ECM component that is frequently leveraged to prepare hydrogels owing to its limited immunogenicity excellent biocompatibility, superior biodegradability, and ability to facilitate cell adhesion [99]. Noyes et al. [100] explored the use of human collagen as a spacer with the goal of enhancing the distance between the prostate and the anterior rectal wall by injecting 20 mL of human collagen into the perirectal space via the perineal route in 11 patients undergoing radiotherapy. This resulted in an ~50% decline in the average radiation dose delivered to the anterior rectal wall without any significant rectal toxicity, and this therapeutic intervention was well tolerated. Owing to the tendency of collagen to form clumps, however, achieving a desirable consistency when manipulating large volumes was challenging. Commercially available human collagen must generally be reconstituted using sterile saline. As collagen hydrogels are homogeneous and injectable, they are well-positioned to overcome these limitations associated with more traditional collagen preparations. However, no studies to date have explored the use of collagen hydrogels as spacers during radiotherapy, highlighting an interesting direction for further investigation.

### 3.4. Other Radiation-Induced Diseases

Radiation-induced lung injury (RILI) can develop following the radiotherapeutic treatment of thoracic tumors owing to the high sensitivity of pulmonary tissue to ionizing radiation. RILI typically entails a combination of early radiation pneumonitis followed by radiation-induced pulmonary fibrosis at later time points. The ability to reduce inflammation and fibrotic activity within the lungs is essential to the effective treatment of lung injuries [101,102]. The ECM regulates cellular growth, differentiation, and phenotypic maintenance, while recruiting tissue-specific progenitor cells to support the regeneration of damaged tissues, and thus plays a central role in shaping cellular behaviors and determining the outcomes of remodeling processes [27,103]. The ECM of a given target organ can be solubilized and subsequently manipulated to form hydrogels which exhibit up to 10-fold better regenerative activity as compared to commercial hydrogels composed of biocompatible factors such as gelatin, collagen, and alginate. For example, Zhou et al. [42] designed a lung ECM-based hydrogel by combining lung-derived ECM powder and a hydrochloric acid-pepsin solution, yielding a hydrogel comprised of a fibrous network of growth factors, proteoglycans, and structural proteins that mimicked the chemical and structural properties of native lung tissue. These ECM hydrogels exhibited excellent histocompatibility and cytocompatibility, alleviating radiation-induced lung damage and edema. Given the adverse effects that RILI can have on the quality of life of affected patients, there is a clear pressing need for the further design of hydrogel-based radioprotective interventional strategies capable of treating this debilitating condition.

**Figure 5:**
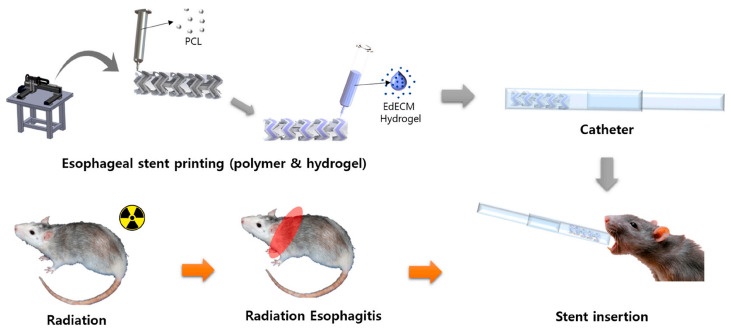
Schematic illustration of the esophagus-derived decellularized extracellular matrix (EdECM) hydrogel-loaded stent. Reprinted with permission from [105].

The value of ECM-based hydrogels as tools for the treatment of radiation-related damage to the esophagus has also been explored. Radiation-induced esophageal disease is most commonly observed in thoracic tumor patients undergoing radiotherapy, and it can cause symptoms including heartburn, chest pain, and dysphagia. No cures for radiation esophagitis have been developed to date, although two major interventional strategies have been explored, including stent insertion aimed at opening narrow regions of the esophagus to reduce dysphagia symptoms as well as supportive treatments including fasting, antibiotics, and parenteral nutrition [104]. The delivery of therapeutic drugs or bioactive compounds within the esophagus has also been identified as an effective means of mitigating inflammation within this compartment. Ha et al. [105] were able to successfully design an esophageal ECM-derived hydrogel and to employ a 3D printing approach to fabricate an esophageal ECM hydrogel-loaded stent. When these stents were placed in the esophagus of model animals, they exhibited excellent biofunctional properties, rheological properties, and physical stability such that they may function as an ideal microenvironmental setting for tissue development. Scaffolds exhibit structural stability and are capable of protecting loaded hydrogels during delivery. The use of a scaffold platform to deliver esophageal ECM hydrogels in vivo was sufficient to rapidly alleviate inflammation and to thereby establish a pro-regenerative tissue microenvironment (Figure 5). Prolonged esophageal exposure to radiation can also cause esophageal fibrosis to occur. In an effort to combat this in a mouse model of radiation-induced esophageal fibrosis, Kim et al. [106] designed a catechol-modified HA hydrogel into which they encapsulated MSC-SPs. Their injectable hydrogel effectively delivered these stem cells to the esophagus and supported their paracrine functionality. The catechol modification of these hydrogels was additionally conducive to tissue adhesion and transplanted cell retention, while the HA-rich hydrogel microenvironment facilitated MSC bioactivation. Table 4 lists the main data on animal models of other diseases induced by radiation treated using hydrogel materials.

## 4. Conclusions and Future Prospects

The present review offers a systematic overview of approaches to the design of radioprotective hydrogels through the use of different radioprotective compounds and rational design strategies with the goal of preventing or treating radiation-related diseases. However, this research still remains in its relatively early stages, and there remain many barriers to the clinical application of these hydrogels which are discussed below.

(1) Hydrogel biosafety is a key consideration when seeking to deploy them in vivo. While most hydrogel materials analyzed to date have exhibited good biosafety profiles in short-term studies, long-term analyses of the risks associated with their use remain to be conducted. Efforts to definitively and comprehensively assess long-term outcomes associated with hydrogel materials are thus vital to their more widespread use as radioprotective tools in clinical settings.

(2) The precise mechanisms whereby radiation exposure can cause normal tissue damage require further study given the inherent complexity and multifactorial nature of radiation-related diseases, which can entail a range of biochemical, cellular, and molecular alterations including altered signal transduction, immune dysregulation, inflammation, and microenvironmental changes. For example, in the context of radiation-induced gastrointestinal disease, factors thought to contribute to pathogenic outcomes include both direct damage to epithelial cells and the local microvasculature as well as neuroimmune interactions, immune system activity, and interactions between intestinal microbes and the local microenvironment [107]. By seeking to better clarify these pathogenic drivers of radiation-related injury, researchers will be able to better guide the development of novel drug capable of targeting these specific mechanisms, thereby improving patient outcomes.

(3) Research focused on using hydrogels to prevent or treat many common radiation-related diseases is lacking at present. For example, while radiation-related ophthalmic diseases including cataracts, retinopathy, glaucoma, and vascular diseases are frequently encountered in the clinic, yet there have been no reports of using hydrogels to treat these conditions [108]. Given that excessive ROS production is the primary driver of radiation-induced eye damage, published works focused on treating oxidative stress-related damage to the eye with hydrogels may provide a valuable basis for the adaptation of these materials as tools to treat radiation-related ocular disease. Grumetto et al. [109], for example, have generated a novel recombinant manganese SOD-containing gel capable of readily eliminating O2^•−^ and other free radicals to protect against oxidative stress-driven damage. The application of their gel preparation prior to ultraviolet radiation exposure effectively protected the eyes of rabbits, thereby protecting them against damage. Radiation-related diseases of the heart, kidneys, spleen, liver, and other tissues are also frequently detected in the clinic. ROS-responsive hydrogels are gradually emerging in different fields, such as myocardial injury, nerve injury, etc. [20,21]. Zhao et al. [110] developed a ROS-responsive hydrogel to remove ROS produced by wound infection. However, there is no report of ROS-responsive hydrogel used for radiation-induced damage, and its effect on radiation induced damage remains to be explored. While the unique properties of hydrogels make them particularly valuable for application to tissue surfaces, ECM-based hydrogels specifically developed for these internal organs may also offer therapeutic value given the promising results observed when using matrix hydrogels comprised of lung and esophageal tissue discussed above. There is thus ample opportunity for the design of novel radioprotective hydrogels in the future.

(4) There is clear value in promoting the further development of hydrogels for the clinical treatment of radiation-induced injury. While a range of promising preclinical studies focused on this topic have been published to date, clinical studies are lacking and those that have been published are primarily case reports or relatively small clinical trials. It is thus vital that other research teams work to develop novel hydrogel materials and to facilitate their translation into clinical settings in order to improve patient outcomes.

## Figures and Tables

**Table 1:** Main data on animal models of RISI treated using hydrogel materials.

Study	Hydrogel Type	Characteristics	Stowage	Function	Model Type	Radiation Type
Zhao 2021 [37]	NaHA hydrogels	Unique viscoelasticity, good water retention, tissue compatibility, and non-immunogenicity	Fullerenols	Protect epidermal stem cells	RISI in rat	Electron rays (6 MeV)
Zhang 2021 [38]	Alginate/HA/polysine hydrogels	Anti-biofouling and antioxidant	Curcumin and epigallocatechin gallate	Alleviate inflammation, scavenge ROS, and promote angiogenesis.	RISI in rat	X-ray (40 Gy)
Nie 2022 [41]	Self-assembled peptide hydrogel	Biodegradable and biocompatible, bioadhesion properties	Gingiva-derived MSCs	Improve wound healing	RISI in rat	^137^Cs γ-ray (50 Gy)
LEE 2017 [58]	Small intestinal dreived-ECM hydrogel	Biocompatibility	Human umbilical cord blood-derived-MSCs	Enhanced wound healing and the angiogenesis	Combined radiation-wound in rat	X-ray (5 Gy)
Park 2018 [61]	Chitosan microparticlepluronic F127 hydrogel	Thermo-responsive	Substance P and transforming growth factor-β1	Accelerates regenerative wound repair	RISI in rat	^60^Co γ-ray (40 Gy)
Kulshrestha 2020 [62]	Citrate-Based Hydrogel	Combined property of lipid based drug delivery	Sildenafil	Improve delayed wound healing	RISI in rat	^60^Co γ-ray (45 Gy)
Hao 2022 [63]	Graphene oxide/Sodium alginate Based(GO/SA) hydrogels	Biocompatibility, antibacterial, radioresistance, sprayable	Interferon-alpha inducible protein 6, Polydopamine	Improve inflammation and induce granulation tissue formation, angiogenesis	RISI in rat	Electron rays (6 MeV)

Abbreviation: ECM—extracellular matrix; MSCs—mesenchymal stem cells; RISI—Radiation-induced skin injury; HA—hyaluronic acid.

**Table 2:** Main data on RISI in humans treated using hydrogel materials.

Study	Research Type	Patients Type	Number of Patients	Hydrogel Type	Characteristics of Hydrogel	Controls	Resuts
Aysan 2017 [64]	RCT	BC	47	Boron-based gel	Antioxidant properties	Placebo	Positive
Tungkasamit 2022 [65]	RCT	HNC	120	Aloe vera gel	Anti-inflammatory	Placebo gel	Positive
Heggie 2002 [67]	RCT	BC	225	Aloe Vera Gel	Anti-inflammatory and antibacterial.	Aqueous cream	Negative
Gollins 2008 [68]	RCT	HNC or BC	30	Cross-linked polyethylene oxide hydrogel	NA	Gentian violet	Positive
Kouloulias 2013 [69]	Single arm study	BC	30	Sucralfate gel	Promotes angiogenesis, anti-inflammatory	Historical controls	Positive
Iacovelli 2017 [70]	Single arm study	HNC	41	Hyaluronic acid gel (Xonrid^®^)	Hygroscopic moisturizing effect; emollient, softening, nourishing properties.	Historical controls	Positive
Ahn 2020 [71]	RCT	BC	56	Silicone gel(Strata XRT^®^)	Promote a moist wound-healing environment.	Moisturizing cream(X-derm^®^)	Positive
Ferreira 2020 [72]	RCT	HNC	48	Chamomile gel	Anti-inflammatory	Cream of urea	Positive
Sahin 2022 [73]	RCT	BC	257	Boron-Based Gel	NA	Placebo gel	Positive

Abbreviation: RCT—randomized controlled studies; HNC—head and neck cancer; BC—breast cancer; NA—not available.

**Table 3:** Main data on animal models of ORN treated using hydrogel materials.

Study	Hydrogel Type	Characteristics	Stowage	Model Type	Evaluation Indicator	Radiation Type
Jin 2015 [84]	HA-based hydrogel	MMP-sensitive,	Rat MSCs and bone morphogenetic protein-2	Osteoradionecrosis in the rat mandible	BV and BMD	X-ray(30 Gy)
Park 2017 [85]	Matrigel^®^ matrix	Promote bone regeneration	Tonsil-derived MSCs	Osteoradionecrosis in a rat model	BMD, BV, BV/TV, and TTV	X-ray(20 Gy)
Ehrhart 2005 [86]	Gelatin hydrogel	Localized and sustained drug release	Transforming growth factor-β1	Irradiated long-bone defects in rabbit	The amounts of bone formation	^60^Co γ-ray (50 Gy)

Abbreviation: ORN—Osteoradionecrosis; HA—hyaluronic acid; MMP—matrix metalloproteinase; MSCs—mesenchymal stem cells; BV—Bone volume; BMD—bone mineral density; TV—total volume; TTV—trabecular thickness values.

**Table 4:** Main data of hydrogel materials treated on animal models of other disease induced by radiation injury.

Study	Hydrogel Type	Characteristics	Mode of Presentation	Stowage	Model Type	Evaluation Indicator
Zhou 2019 [42]	Lung tissue ECM hydrogel	Affect cell behavior and influence remodeling outcomes	Endotracheal injection	-	Radiation-induced lung injury in mice	Lung histopathology injury and pulmonary edema
Ha 2021 [105]	Esophagus-derived dECM hydrogel	Promote tissue regeneration	Loaded in esophageal stents	-	Radiation esophagitis rat model	Histological morphology and inflammatory responses
Kim 2021 [106]	HA hydrogel	Promote tissue adhesion and cell retention	Esophagus injection	Human mesenchymal stem-cell spheroids	Radiation-induced esophageal fibrosis in rat model	Histological morphology and inflammatory responses

Abbreviation: ECM—extracellular matrix; HA—hyaluronic acid.

## Data Availability

Not applicable.
